# Discrete Bat Algorithm for Optimal Problem of Permutation Flow Shop Scheduling

**DOI:** 10.1155/2014/630280

**Published:** 2014-08-27

**Authors:** Qifang Luo, Yongquan Zhou, Jian Xie, Mingzhi Ma, Liangliang Li

**Affiliations:** College of Information Science and Engineering, Guangxi University for Nationalities, Nanning, Guangxi 530006, China

## Abstract

A discrete bat algorithm (DBA) is proposed for optimal permutation flow shop scheduling problem (PFSP). Firstly, the discrete bat algorithm is constructed based on the idea of basic bat algorithm, which divide whole scheduling problem into many subscheduling problems and then NEH heuristic be introduced to solve subscheduling problem. Secondly, some subsequences are operated with certain probability in the pulse emission and loudness phases. An intensive virtual population neighborhood search is integrated into the discrete bat algorithm to further improve the performance. Finally, the experimental results show the suitability and efficiency of the present discrete bat algorithm for optimal permutation flow shop scheduling problem.

## 1. Introduction

Scheduling problems are taking the very important effect in both manufacturing systems and industrial process for improving the utilization efficiency of resources [1], such as, aircraft landing scheduling problem, job shop scheduling problem, and flow shop scheduling problem. In the past several decades, scheduling problems are widely researched. Permutation flow shop scheduling problem (PFSP) is one of best known production scheduling problems, which can be viewed as a simplified version of the flow shop problem and has been proved that non-deterministic polynomial (NP) time [2]. Due to its significance in both academic and engineering applications, the permutation flow shop with the criterion of minimizing the makespan, maximum lateness of jobs, or minimizing total flow time, a great diversity of methods have been proposed to solve PFSP and some achievements were obtained.

So far, there are many methods that have been introduced for solving PFSP with the objective of minimizing the makespan. To sum up, these methods can be classified into three categories: exact methods, constructive heuristic methods, and metaheuristic algorithms based on the constructive operation and neighborhood search. Exact methods include branch and bound method [3], integer linear programming method [4], and so on. Constructive heuristic methods which build some rule to construct a feasible scheduling, such as,

Johnson method, Rajendran NEH can be viewed as the typical cases [5]. Among them, the NEH is one of the most successful constructive methods and can provide comparable results with metaheuristics. The metaheuristics mainly include genetic algorithm (GA) [6], particle swarm optimization algorithm (PSO) [7], differential evolution (DE) [8], and bat algorithm (BA) [9] and so on. Many metaheuristic algorithms are used to solve flow shop scheduling based on the constructive operation and neighborhood search in the past few years. In [6], Wang and Zheng proposed a SGA to solve flow shop scheduling, which used the well-known NEH combined with GA to generate the initial population and applied multicrossover operators to enhance the exploring potential. In [10], Tasgetiren et al. applied the PSO algorithm to solve PFSP for makespan and total flow time minimization by using the smallest position value rule borrowed from the random key representation of GA, and the proposed algorithm was combined with the variable neighborhood-based local search, as called PSO_VNS. Liu et al., in [11], proposed an efficient particle swarm optimization based mimetic algorithm (MA) for PFSP to minimize the maximum completion time. In [12], two effective heuristics are used during the local search to improve all generated chromosomes in every generation. Yagmahan and Yenisey have proposed a multiobjective ant colony system algorithm to simultaneously minimize objectives of makespan and total flow time [13]. Tasgetiren et al. present a discrete artificial bee colony algorithm hybridized with a variant of iterated greedy algorithms to find the permutation that gives the smallest total flow time [14]. In [15], a novel mechanism is employed in initializing the pheromone trails based on an initial sequence, and the pheromone trail intensities are limited between lower and upper bounds which change dynamically. Moreover, a local search is performed to improve the performance quality of the solution. In [16], Li and Yin applied a differential evolution based memetic algorithm, named ODDE, to solve PFSP by combining with NEH heuristic initialization, opposition-based learning, pairwise local search, and fast local search in ODDE. In [17], Liu et al. a multipopulation PSO based memetic algorithm for permutation flow shop scheduling is proposed. In [18], Mirabi proposed a novel hybrid genetic algorithm to solve the sequence-dependent permutation flow shop scheduling problem. In [19], Victor and Framinan use on insertion tie-breaking rules in heuristics for the permutation flow shop scheduling problem.

In recent years, a bat algorithm (BA) as a new metaheuristic optimization algorithm is proposed [9]. BA is inspired by the intelligent echolocation behavior of microbats when their foraging. After the bat algorithm is proposed by Yang in 2010, bat algorithm is used to solve various optimization problems. For example, Gandomi et al. focus on solving constrained optimization tasks [20]. Yang and Gandomi apply bat algorithm to solve many global engineering optimizations [21]. Mishra et al. present a model for classification using bat algorithm to update the weights of a functional link artificial neural network (FLANN) classifier [22]. Meanwhile, there are improved bat algorithms that are applied to various optimization problems; Xie et al. proposed a DLBA bat algorithm based on differential operator and Lévy flights trajectory to solve function optimization and nonlinear equations [23]. Wang et al. proposed a new bat algorithm with mutation (BAM) to solve the uninhabited combat air vehicle (UCAV) path planning problem [24]. In this paper, we propose a discrete bat algorithm (DBA) to solve PFSP. Here, the DBA is constructed based on the idea of continuous bat algorithm, which divide whole scheduling problem into many subscheduling problems, then NEH heuristic was introduced to solve subscheduling problem. Moreover, some subsequences are operated with certain probability in the pulse emission and loudness phases. An intensive virtual population neighborhood search is integrated into the DBA to further improve the performance. Finally, the experimental results show the effectiveness of the discrete bat algorithm for PFSP.

## 2. Problem Descriptions and Bat Algorithm

### 2.1. Permutation Flow Shop Scheduling Problem

The permutation flow shop scheduling problem (PFSP) in the paper consists of a set of jobs on a set of machines with the objective of minimizing the makespan. In PFSP, *n* jobs are to be processed on a series of *m* machines, sequentially. All jobs are processed in the same permutation; meanwhile, every job is processed in one machine only once and each machine can only process one job at a time, and all jobs are processed in an identical processing order on all machines.

The permutation flow shop scheduling problems are often denoted by the symbols *n*∣*m*∣prmu∣*C*_max⁡_, where *n* represents the number of jobs; *m* is the number of machines; prmu denotes the type of flow shop scheduling problem; and *C*_max⁡_ is the makespan. Let *t*_*i*,*j*_ (1 ≤ *i* ≤ *n*,  1 ≤ *j* ≤ *m*) be the times of job *i* processed on machine *j*, assuming preparation time for each job is zero or is included in the processing time *t*_*i*,*j*_; *π* = (*j*_1_, *j*_2_,…, *j*_*n*_) is a scheduling permutation of all jobs. Π is set of all scheduling permutation. *C*(*j*_*i*_, *k*) is completion time of job *j*_*i*_ on machine *k*, and every job will be processed on machine 1 to machine *m* orderly. The completion time of the permutation flow shop scheduling problem according to the processing sequence *π* = (*j*_1_, *j*_2_,…, *j*_*n*_) is shown as follows:

(1) C(j1,1)=tj1,1,C(ji,1)=C(ji−1,1)+tji,1, i=2,3,…,n,C(j1,k)=C(j1,k−1)+tj1,k, k=2,3,…,m,C(ji,k)=max⁡⁡{C(ji−1,k),C(ji,k−1)}+tji,k,i=2,3,…,n, k=2,3,…,m,π∗=arg{Cmax⁡(π)=C(jn,m)}⟶min⁡, ∀π∈Π,

where *π*_∗_ is the most suitable arrangement which is the goal of the permutation flow shop problem to find *C*_max⁡_(*π*_∗_) is the minimal makespan.

### 2.2. Bat Algorithm (BA)

The bat algorithm (BA) is an evolutionary algorithm first introduced by Yang in 2010 [9]. In simulations of BA, under several ideal rules, the updated rules of their positions *x*_*i*_ and velocities *v*_*i*_ in a D-dimensional search space are defined. The new solutions *x*_*i*_^*t*^ and velocities *v*_*i*_^*t*^ at generation *t* are given by

(2) fi=fmin⁡+(fmax⁡−fmin⁡)β,vit=vit−1+(xit−x∗)fi,xit=xit−1+vit,

where *β* ∈ [0,1] is a random vector drawn from a uniform distribution, *f*_*i*_ denotes frequency of each bat, and the frequency *f*_*i*_ ∈ [*f*_min⁡_, *f*_max⁡_]. Here *x*_∗_ is the current global best location (solution) which is located after comparing all the solutions among all the *n* bats.

After the position updating of bat, a random number is generated; if the random number is greater than the pulse emission rate *r*_*i*_, a new position will be generated around the current best solutions, and it can be represented by

(3) $$\begin{array}{c} x=x_{*}+\varepsilon A_{t}, \end{array}$$

where *ε* ∈ [−1,1] is a random number, while *A*_*t*_ = 〈*A*_*i*_^*t*^〉 is the average loudness of all the bats at current generation *t*.

Furthermore, the loudness *A*_*i*_ and the pulse emission rate *r*_*i*_ will be updated and a solution will be accepted if a random number is less than loudness *A*_*i*_ and *f*(*x*_*i*_) < *f*(*x*_∗_). *A*_*i*_ and *r*_*i*_ are updated by

(4) $$\begin{array}{c} A_{i}^{t+1}=\alpha A_{i}^{t},\;\;r_{i}^{t+1}=r_{i}^{0}\left[1-\exp\left(-\gamma t\right)\right], \end{array}$$

where *α*, *γ* are constants and *f*(·) is fitness function. The algorithm repeats until the termination criterion is reached. The basic steps of the bat algorithm (BA) can be described in Algorithm 1.

## 3. Discrete Bat Algorithm for PFSP

Since standard BA is a continuous optimization algorithm, the standard continuous encoding scheme of BA cannot be used to solve PFSP directly. Meanwhile, many combinational optimization problems are discrete problem, and PFSP is a typical case. In order to apply BA to PFSP, there are two methods: the first method is to solve PFSP using continuous BA, however, this method needs to construct a direct mapping relationship between the job sequence and the vector of individuals in BA; the second method is to construct a discrete BA for PFSP. Therefore, in this paper, a discrete bat algorithm is proposed to solve PFSP with minimal makespan.

**Figure 1:**
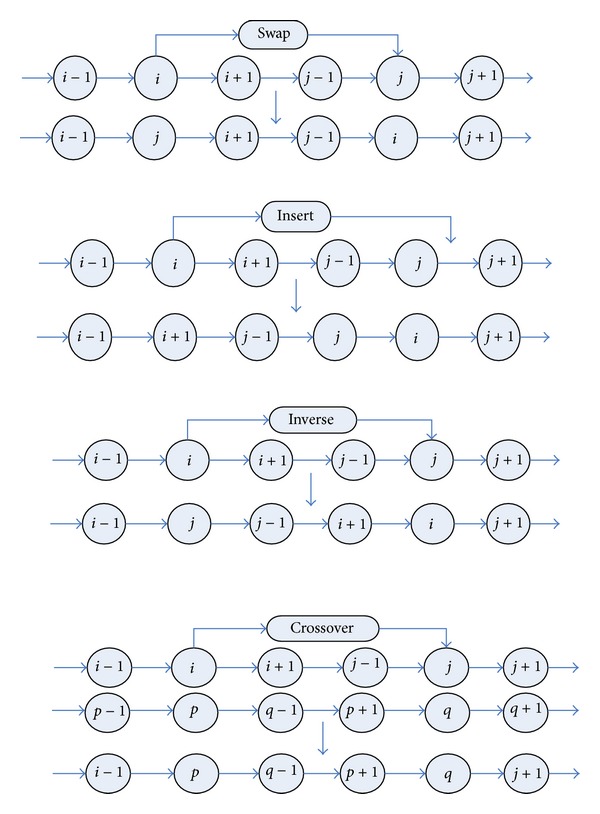
Four neighborhood operations (swap, insert, inverse, and crossover).

In addition, for PFSP, some neighborhood search methods always are used to enhance the quality of the solution, and the performance is remarkable. In this paper, four neighborhood search methods, that is, insert, swap, inverse, and crossover, will be employed. These neighborhood operations are shown in Figure 1. The details of these neighborhoods are as follows.

*Swap.* Choose two different positions from a job permutation randomly and swap them. 

*Insert.* Choose two different positions from a job permutation randomly and insert the back one before the front. 

*Inverse.* Inverse the subsequence between two different random positions of a job permutation. 

*Crossover.* Choose a subsequence in a random interval from another random job permutation and replace the corresponding part of subsequence.

### 3.1. Solution Representation in DBA

In original BA, the position of each virtual bat is viewed as a candidate solution of problem; these bat individuals adjust the flight speed by randomly selecting frequency of sonic wave which they emitted and then update the position of bats. Furthermore, the pulse emission rate and loudness are used to control the intensive local search that is process to generate a new individual around the current global best solution. In DBA, in general, the position *x*_*i*_^*n*^(*t*) of individual *i* denotes a scheduling plan on *t*th iteration, where *n* represents the scheduling plan including *n* jobs. The *x*_*i*_^*n*^(*t*) is also viewed as a *π* = (*j*_1_, *j*_2_,…, *j*_*n*_). For example, if *x*_1_^4^(2) = [3  2  1  4], which represents the processing order of all jobs on all machines, is 3 → 2 → 1 → 4, this permutation represents the position of first bat individual in second generation. The velocity *v*_*i*_^*N*^(*t*) consists of a part of scheduling plan or whole scheduling plan on *t*th iteration, where *N* ≤ *n*.

### 3.2. Population Initialization

In this paper, the DBA is applied to explore the new search space. Initial swarm is often generated randomly, and, in DBA, this initial strategy is adopted. Meanwhile, recent studies have confirmed the superiority of NEH over the most recent constructive heuristic [5]. Many metaheuristic algorithms in order to generate an initial population with certain quality and diversity take advantage of the NEH heuristic to generate some individuals and the rest of the individuals are initialized with random values [16]. In this paper, this kind of initialization strategy is not including in DBA, but NEH is used in position updating of bat. However, a discrete bat algorithm with NEH initialization strategy is experimented. By experiments, we find that the combination of NEH initialization strategy and succeeding operation always deteriorates the population diversity, by tracking offspring, the results showed that all the individuals in the final population were similar.

In [25], NEH heuristic is regarded as the best heuristic for the PFSP. The NEH algorithm is based on the idea that the high processing time on all machines should be scheduled as early in the sequence as possible. The NEH heuristic has two phases.

1. The jobs are sorted in nonincreasing sums of their processing time.
2. A job sequence is established by evaluating the partial schedules based on the initial order of the first phase. The standard NEH and a variant of standard NEH (NEH1) can be described as shown in Algorithm 2; the only difference of two NEH is that the inserted position of new job in partial schedules is different: NEH1 have only two possibilities of inserting.

### 3.3. Position Updating of Bat

Scheduling problem with many jobs can be viewed as a combination of many subscheduling problems; as we all know, we can apply dynamic programming to solve this problem. However, in this paper, the idea of partition is adopted, a complete scheduling sequence is divided into many segments, and each subscheduling problem is solved by superior NEH.

In continuous BA, the bat individual randomly selects a certain range of frequency, and its speed is updated according to their selected frequency; at last, a new position is generated using its speed and its own position. In DBA, for each individual, firstly, a frequency *f* is selected in the range of frequency [*f*_min⁡_, *f*_max⁡_]; frequency *f* denotes the number of subsequences, where *f*_min⁡_, *f*_max⁡_ are two integers in the range of job amount *n*,

(5) $$\begin{array}{c} f=\left\lfloor f_{\max}+\left(f_{\min}-f_{\max}\right)\times \left(\frac{t}{t_{\max}}\right)\right\rfloor , \end{array}$$

where ⌊·⌋ denotes rounded down function. Secondly, frequency *f* decides the starting location and ending location of each subsequence, and the position *x*_*i*_^*n*^(*t*) is divided into *f* subsegments; these subsequences are viewed as the velocity *v*_*i*,*f*_^*N*^(*t*) of bat individual, where *N* ≤ *n*. Thirdly, these velocities are updated by NEH; the new velocity is called *v*_tmp,*f*_^*N*^(*t*). At last, the corresponding part of *x*_*i*_^*n*^(*t*) is replaced by *v*_tmp,*f*_^*N*^(*t*). In order to facilitate understanding, there is a simple instance: *f* = 3 ∈ [2,4], *n* = 8, *x*(*t*) = [5,1, 3,2, 4,7, 6,8]; *v*_1_ = [5,1, 3], *v*_2_ = [2,4, 7], *v*_3_ = [6,8]; *v*_tmp,1_ = [1,3, 5], *v*_tmp,2_ = [4,2, 7], *v*_tmp,3_ = [6,8]; *v*_*i*_^*N*^(*t*) = [2,1, 3], so *x*(*t* + 1) = [1,3, 5,4, 2,7, 6,8].

### 3.4. Pulse Emission Rate Local Operation

In original BA, the pulse emission rate and loudness are used to control the intensive local search, that is to generate a new individual around the current global best individual *gbest*_*x*. In DBA, each individual has its own pulse emission rate *r*_*i*_. The initial pulse emission rate is a positive and smaller number; with the increase of iteration, pulse emission rate *r*_*i*_ will increase to 1. The updating of *r*_*i*_ using

(6) $$\begin{array}{c} r_{i}\left(t\right)={1+\exp\left(-\frac{10}{t_{\max}}\times \left(t-\frac{t_{\max}}{2}\right)+r_{i}\left(1\right)\right)}^{-1}. \end{array}$$

**Figure 2:**
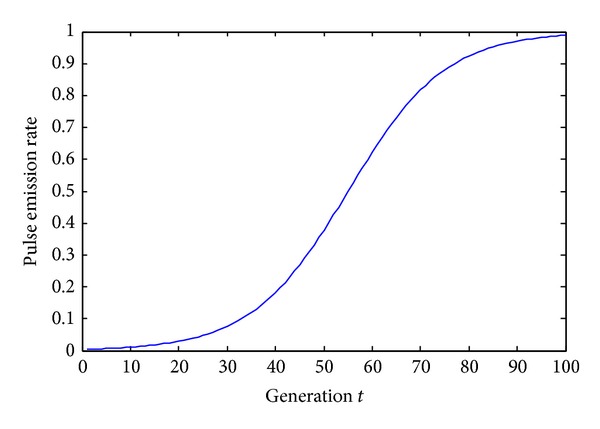
Updating curve of pulse emission rate *r*_*i*_.

Figure 2 presents an example of updating curve of pulse emission rate *r*_*i*_ under maximal iterations is 100, pulse emission rate *r*_*i*_ has a value ranging from 0 to 1. Using this updating formula, the algorithm can not only quickly exploit near the current optimal position in the early iteration, so that speed up the convergence rate, but also can mainly concentrate in diversity in later search and can avoid to fall into local optima.

The pulse emission rate *r*_*i*_ will control the subsegment local operation. For each individual, randomly generate a random number; if this random number is larger than its *r*_*i*_, this position of bat individual will be updated by random swap two segments defined by frequency *f*; otherwise, the updating operation will be implement by random inserting operation; the pseudo code can be described as shown in Algorithm 3.

### 3.5. Loudness Local Operation

In DBA, the loudness Ld_*i*_ of bat individual *i* is relative to its own fitness fit_*i*_; the better fitness, the less loudness. The loudness can be described by

(7) $$\begin{array}{c} {\text{Ld}}_{i}=\frac{\left({\text{fit}}_{i}-{\text{fit}}_{\min}\right)}{\left({\text{fit}}_{\max}-{\text{fit}}_{\min}\right)}, \end{array}$$

where fit_*i*_ is the fitness of individual *i* and fit_min⁡_ and fit_max⁡_ are the minimum and maximum fitness in current population, respectively. In DBA, the loudness reflects the quality of individual. In this subsection, there are two kinds of local search embedded into algorithm, random subsequence inverse and random subsequence inserting. Note that, where inserting operation is different from inserting operation in Section 3.4.

In this part, for each individual, randomly generate a random number; if this random number is larger than its Ld_*i*_, a random length of subsequence is randomly selected in range of [1, ⌊*D*/2⌋]; this position of bat individual will be updated by inserting operation with random subsequence; otherwise, the updating operation will be implement by random subsequence inverse operation. Note that the subsequence is a portion of the current best position *gbest*_*x*; however, the corresponding replacement portion is the individual *x*_*i*_ in bat population, and the pseudo code can be described as show in Algorithm 4.

Although this inserting and inverse operation may generate invalid scheduling sequence,those invalid scheduling sequences need to adjust to a feasible solution. The adjustment of the pseudo code can be described as show in Algorithm 5.

In order to facilitate understanding, process of adjustment *x* = [3,6, 3,2, 1,5, 5,3], {*S*} = [1,2, 3,5, 6], {*Sid*} = [5,4, 8,7, 2], {*R*} = [4,7, 8], {*Rid*} = [1,3, 6], *IO* = [3,1, 2], and *x*_adjust_ = [8,6, 4,2, 1,7, 5,3].

### 3.6. Intensive Virtual Population Neighborhood Search

In this paper, an intensive virtual population neighborhood search with same population size is easily embedded in DBA for solving PFSP. The purpose of the virtual population neighborhood search is to find a better solution from the neighborhood of the current global best solution. In this part, three neighborhoods, that is, insert, swap, and single-point move backward operate, are employed. These operations are used to improve the diversity of population and enhance the quality of the solution.

In order to enhance the local search ability and get a better solution, a new population is generated based on the current global best solution, and the population size is not less than original bat population; the new population is called virtual population. The new population size ps_1_ = *μ* × ps, *μ* ≥ 1 is real number.

Firstly, the virtual population is generated by randomly selecting two jobs to perform swap operation. Secondly, the virtual population is generated by randomly selecting a job and insert into another random location. At last, the single-point move backward operation is performed also based on current global best individual *gbest*_*x*. In the simulation, first of all, a job position *i* is chosen randomly in *gbest*_*x*; the selected job *i* is inserted into the back of job *i*, orderly, until the population size ps_1_ is reached. For example, the population size ps_1_ = 3, random job position *i* = 2, and *gbest*_*x* = [2,5, 4,1, 3]; the virtual population is generated as follows:

(8) $$\begin{array}{c} \left[\begin{array}{ccccc} 2 & 4 & 5 & 1 & 3 \end{array}\right]\\ \left[\begin{array}{ccccc} 2 & 4 & 1 & 5 & 3 \end{array}\right]\\ \left[\begin{array}{ccccc} 2 & 4 & 1 & 3 & 5 \end{array}\right] \end{array}$$

### 3.7. Discrete Bat Algorithm (DBA)

In DBA, all individuals once the update either in bat population or in virtual population, these individuals will be evaluated and one solution be accepted as the current global best *gbest*_*x* if the objective fitness of it is better than the fitness of the last *gbest*_*x*. The algorithm terminates until the stopping criterion is reached; the DBA algorithm for PFSP can be described in Algorithm 6.

## 4. Numerical Simulation Results and Comparisons

To test the performance of the proposed DBA for the permutation flow shop scheduling, computational simulations are carried out with some well-studied problems taken from the OR-Library (http://people.brunel.ac.uk/~mastjjb/jeb/info.html). In this paper, 29 problems from two classes of PFFSP test problems are selected. The first eight problems are instances Car1, Car2 through to Car8 designed by Carlier [26]. The second 21 problems are instances Rec01, Rec03 through to Rec41 designed by Reeves and Yamada [27]. So far, these problems have been widely used as benchmarks to certify the performance of algorithms by many researchers.

The DBA is coded in MATLAB 2012a, and in our simulation, numerical experiments are performed on a PC with AMD Athlon(tm) II X4 640 Processor 3.0 GHz and 2.0 GB memory. In the experiment, the termination criterion is set as (*n* × *m*/2) × 30 ms maximum computation time. Setting the time limitation in this way allows the much computation time as the job number or the machine number increases. And, this method is also adopted by many researchers, such as Jarboui et al. [28], Ruiz and Stützle [29]. Each instance is independently run 15 times for every algorithm for comparison.

The comparison method adopts BRE, ARE, and WRE to measure the quality of solution by the percentage difference from *C*_∗_; these expressions as follows:

(9) BRE=Cmax⁡best−C∗C∗×100%,ARE=∑i=1n(Cmax⁡i−C∗C∗)×1n×100%,WRE=Cmax⁡worst−C∗C∗×100%,

where *C*_∗_ is the optimal makespan or upper bound value known so far, the makespan of an obtained solution in DBA is *C*_max⁡_, BRE represents the best relative error to *C*_∗_, ARE denotes the average relative error to *C*_∗_, and WRE represents the worst relative error to *C*_∗_. Std denotes the standard deviation of the makespan. These performance measures are employed in our experiments; these results are rounded to the nearest number which contains 2 or 3 digits after the decimal point.

### 4.1. Parameter Analysis

**Figure 3:**
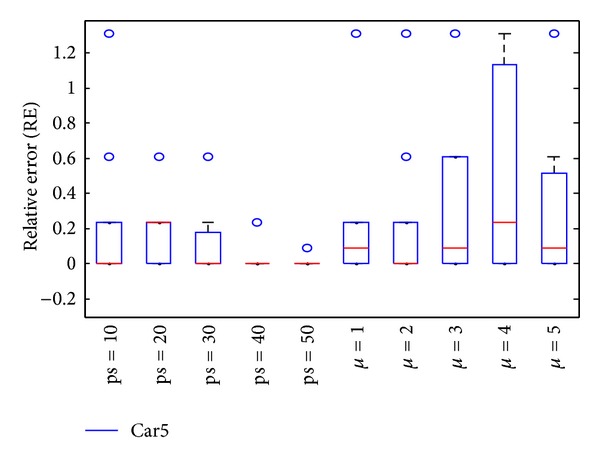
Box-and-whisker diagram of Car5.

**Figure 4:**
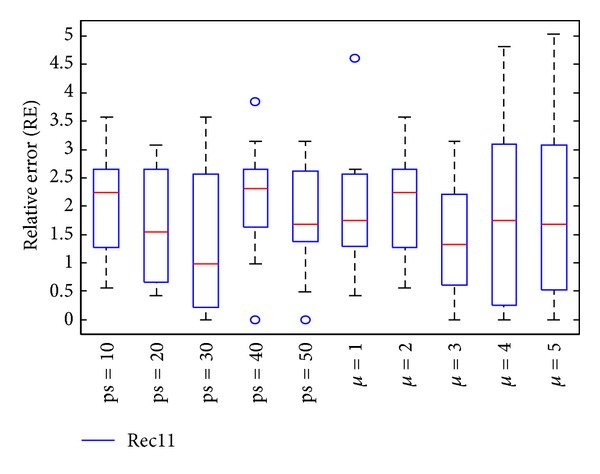
Box-and-whisker diagram of Rec11.

In the subsection, parameters of DBA are determined by experiments, and the impact of each parameter is analyzed. In DBA, parameters ps, *μ* are tested. ps is population size, A small ps may lead insufficient information provided, and the diversity cannot guarantee. On the other side, a large one indicates diversity is sufficient, but the computing time will increase. *μ* determines the size of virtual population; the large one can perform large single point neighborhood search, which may achieve a better solution, especially, the current best solution extraordinarily approximated the exact solution; however, an oversize will increase the computing time, and the precision of optimal solution may have lesser improvement. In order to evaluate the sensitivity of parameters, Car5 and Rec11 are chosen to run 15 times and the results are shown in Figures 3 and 4.

Figures 3 and 4 represent the relative error of test case Car5 and Rec11 after 15 times independent running, which showed the sensitivity of parameters ps and *μ*. *μ* = 2 when test parameter ps, and ps = 10 when test parameter *μ*. From the two test cases, for Car5, the performance is better and better while parameter ps gradually increases. But for Rec11, ps equal to 40 or 50 can achieve exact solution, but the performances do not follow the laws of Car5. In DBA, the parameter ps takes a compromise values, ps = 50. Similarly, parameter *μ* equal to 2 is optimal for Car5; however, *μ* = 3 is optimal for Rec11. In order to balance all test cases, the parameter *μ* is set as 1 while ps = 50.

### 4.2. Comparisons of DBA, DBA_NEH1, and DBA-IVPNS

In order to evaluate the performance of each strategy, two variants of DBA are compared, whose abbreviations are as follows.

1. DBA: DBA with NEH.
2. DBA_NEH1: DBA with NEH1.
3. DBA-IVPNS: DBA without intensive virtual population neighborhood search.

At this group experiment, the parameter setting is ps = 10, *μ* = 2, termination criterion is set as (*n* × *m*/2) × 10 ms maximum computation time, and the algorithm is run 15 times independently. The statistical performances of DBA, DBA _NEH1, and DBA-IVPNS are shown in Table 1.

From Table 1, we can find out that the average performance of DBA is better than the other two variants of DBA; for benchmarks Car1 to Car8, the DBA-IVPNS is better; the reason may be that the IVPNS implementation is single-point operation on the current global best individual *gbest*_*x*; this operation may improve the quality of solution, bur this needs much computing time, so the DBA-IVPNS have more time to explore of more new position. However, from Rec1 to Rec41, the DBA is much better than other variants. For DBA_NEH, only it has a difference that the position updating of bat by NEH1. The NEH1 has lesser computational complexity than NEH. From experiment results, we can find out that DBA_NEH1 can find better solutions for several benchmarks. In general, the DBA is better than DBA_NEH1 for all benchmarks.

**Figure 5:**
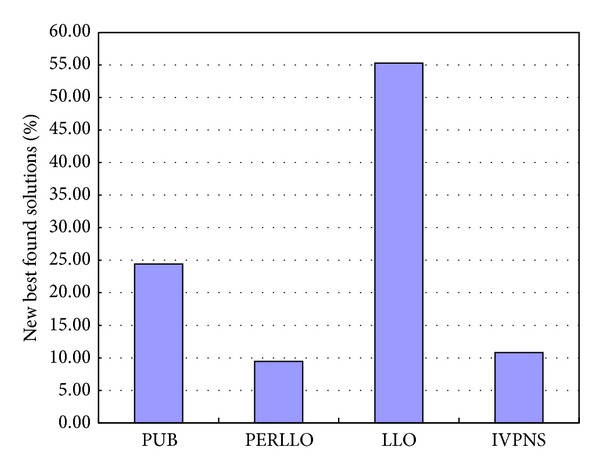
The contribution of each strategy move to finding a new best solution.

In addition, in order to demonstrate the effect of each strategy in the specific scheduling problem, the frequency of finding a new best solution by applying these moves in DBA is recorded; it can show the contribution of each strategy. The Car1 to Car8 and Rec1 to Rec15 16 benchmarks are chosen to tested. Each problem was run 10 times; each time a new best solution was found by the algorithm; the move resulting in this improvement was recorded.  Figure 5 demonstrates the percentage of contribution.

### 4.3. Comparisons of DBA, PSOMA, PSOVNS, OSA

In order to show the effectiveness of DBA, we carry out a simulation to compare our DBA with other state-of-art algorithms, that is, PSOMA proposed by Liu et al. [11], PSOVNS proposed by Tasgetiren et al., and experimental results reference [5], and SA is a simulated annealing, the experimental results reference [16]. The population size is 50 and the termination criterion is set as (*n* × *m*/2) × 30 ms maximum computation time. The experimental results are listed in Table 2.

From Table 2, for the Car problems, the DBA, PSOVNS, PSOMA, and OSA all can find the exact solution, and DBA is better than the other algorithm on ARE. For the Rec problems, DBA also can find better solutions. Compared with DBA, PSOVNS, PSOMA, and OSA, the DBA achieved 14 exact solutions; several optimal job permutations are shown in Table 3. PSOVNS achieved 11 exact solutions, PSOMA achieved 16 exact solutions, and OSA achieved 13 exact solutions. For all test problems, obtained solutions of DBA are not better than the PSOMA and OSA, but the performance is similar to PSOMA and OSA.

In order to compare each norm (BRE, ARE, WRE, and Std) of corresponding algorithms, for all benchmarks, each norm is scored among corresponding algorithms. The first is score 4, the second is score 3, the third is score 2, the fourth is score 1, and the last is score 0, if several results are same, they have same score. The statistical results are listed in Table 4. From Table 4, for Car problems, the DBA is best on ARE, the DBA and PSOMA are identical on WRE, DBA has better Std compared with OSA. For Rec problems, the OSA and PSOMA have better BRE, the DBA is better than PSOVNS, the DBA is better than PSOVNS, OSA, the DBA is also better than PSOVNS on WRE among DBA, PSOVNS, and PSOMA, but the Std is not better than OSA. The DBA is best on ARE for Car1 to Rec29 among DBA, PSOVNS, PSOMA, and OSA, and the Std is better than OSA. On the whole, the achieved solutions of DBA have better quality. For large-scale scheduling problems, the DBA still have the room for improvement; it also is our further work.

**Figure 6:**
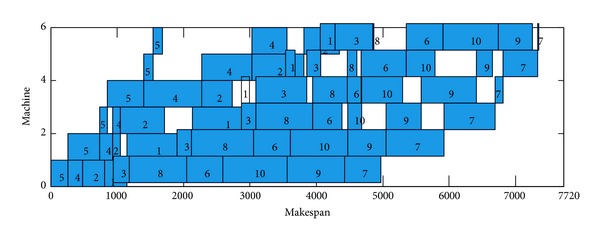
Gantt chart of an optimal schedule for Car05, *π*_∗_ = [5,4, 2,1, 3,8, 6,10,9, 7].

**Figure 7:**
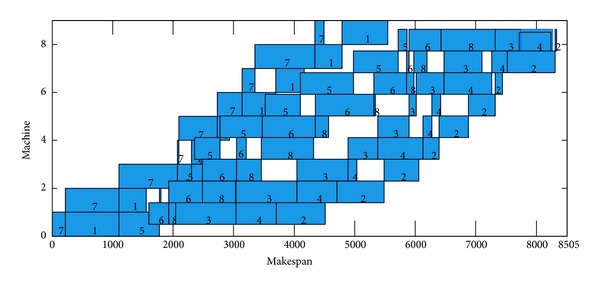
Gantt chart of an optimal schedule for Car06, *π*_∗_ = [7,1, 5,6, 8,3, 4,2].

**Figure 8:**
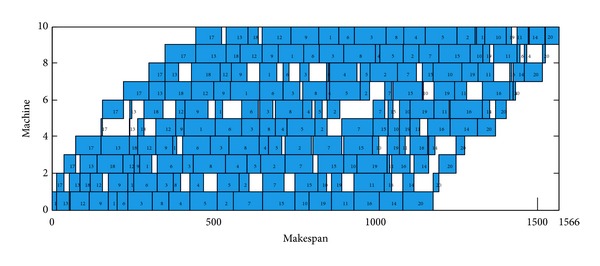
Gantt chart of an optimal schedule for Rec7, *π*_∗_ = [17,13,18,12,9, 1,6, 3,8, 4,5, 2,7, 15,10,19,11,16,14,20].

**Figure 9:**
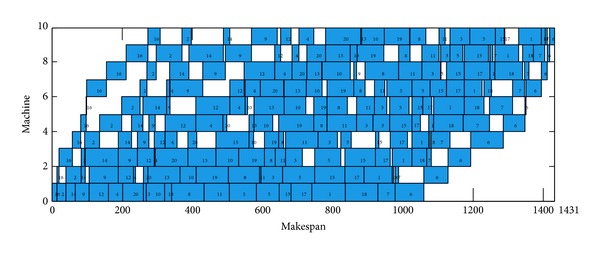
Gantt chart of an optimal schedule for Rec11, *π*_∗_ = [16,2, 14,9, 12,4, 20,13,10,19,8, 11,3, 5,15,17,1, 18,7, 6].

The DBA achieved 14 exact solutions, due to the fact that Rec35 have 10 machines and 50 jobs, the margin of paper is restricted, the Gantt chart of an optimal schedule for Rec35 cannot display on this paper, and the Gantt chart of Car5, Car6, Rec7, and Rec11 is selected as instance. These Gantt charts of an optimal schedule are shown in Figures 6, 7, 8, and 9.

**Figure 10:**
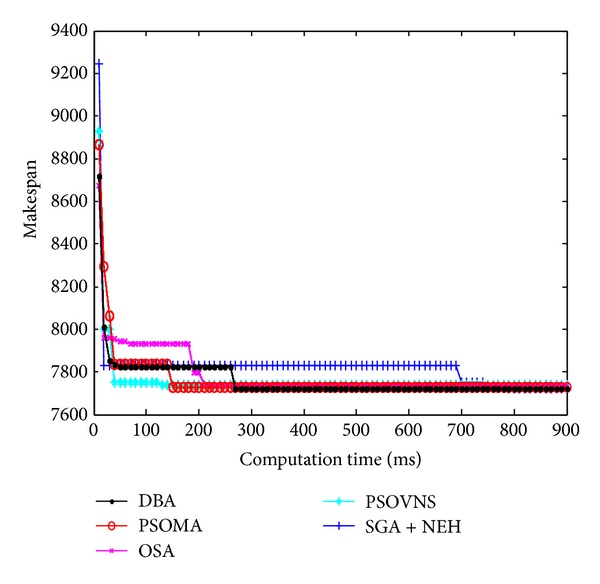
The convergence curves of Car5.

**Figure 11:**
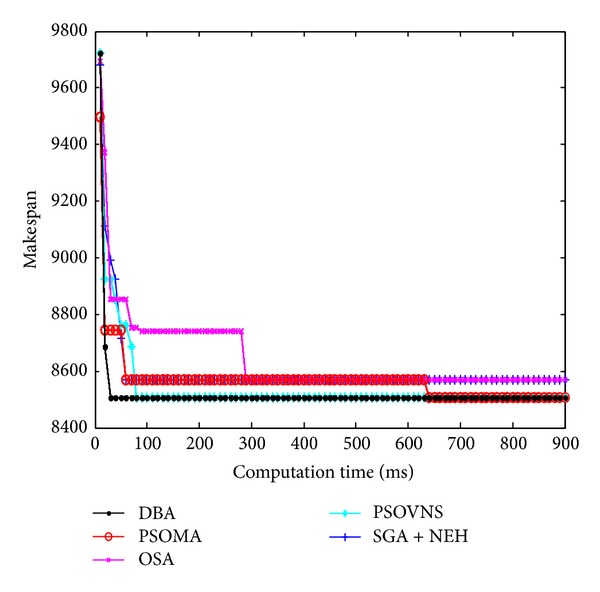
The convergence curves of Car6.

**Figure 12:**
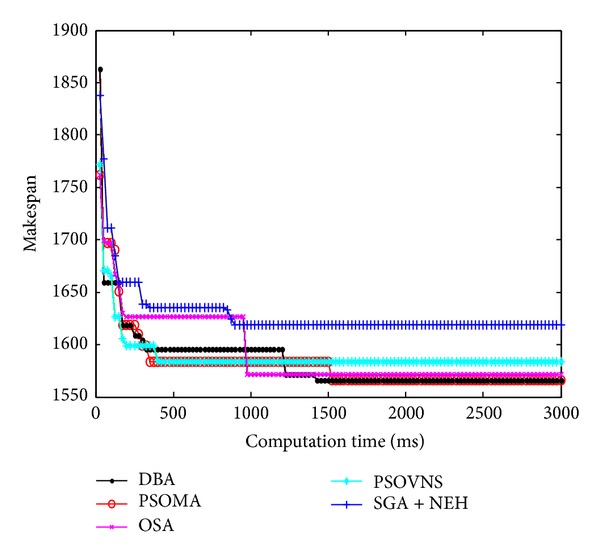
The convergence curves of Rec7.

**Figure 13:**
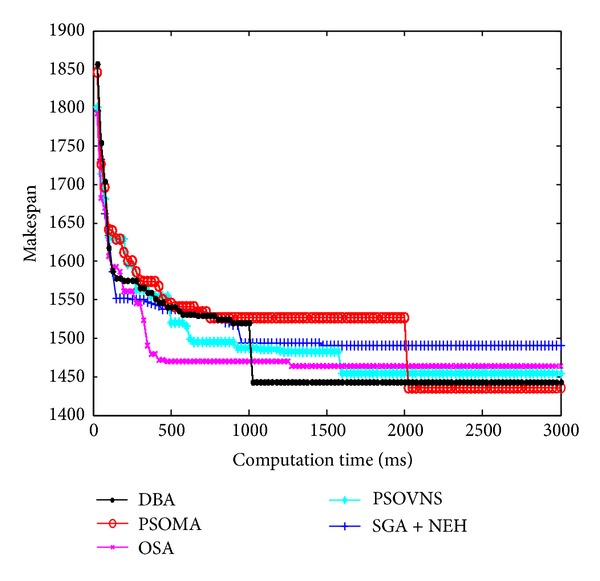
The convergence curves of Rec11.

Figures 10, 11, 12, and 13 show the convergence curves of Car5, Car6 Rec7, and Rec11. From Figures 10 to 13, the convergence rate of DBA is fast, and the precision of solution is prominent. The performance of DBA is similar to PSOMA; however, the convergence rate of DBA is faster than PSOMA in the early phase of iteration. The precision of solution is not as good as PSOMA while the scale of scheduling problems is increasing. The DBA is better than SGA + NEH [5], PSOBNS, and OSA in some aspects.

## 5. Conclusions

In this paper, we construct a direct relationship between the job sequence and the vector of individuals in DBA; a DBA is proposed to solve PFSP. In order to evaluate the performance of the proposed DBA, we compare DBA with several PFSP algorithms with benchmark problems of PFSP. Experimental results have shown that our algorithm is pretty effective, the performance of each strategy is evaluated, and sensitivity of parameters is analyzed. Moreover, our further work is to study the theoretical aspects as well as the performance of the technique.

## Figures and Tables

**Algorithm 1:**
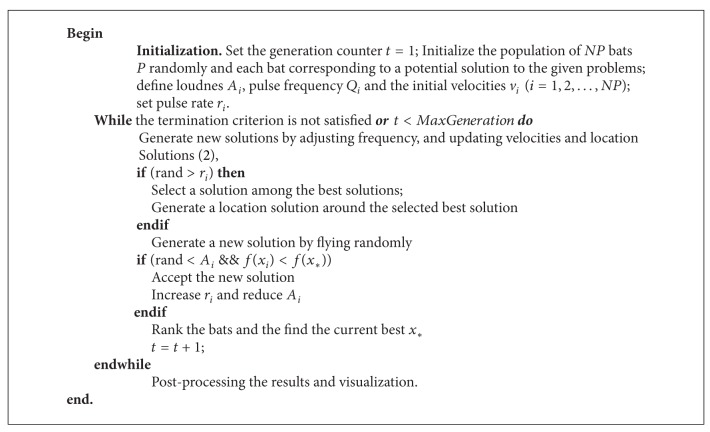
Basic bat algorithm (BA).

**Algorithm 2:**
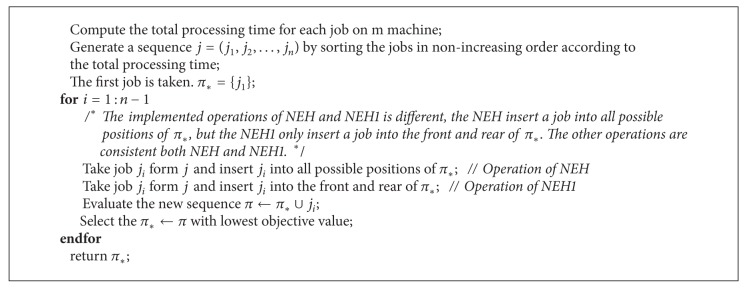
The pseudocode of NEH and NEH1.

**Algorithm 3:**
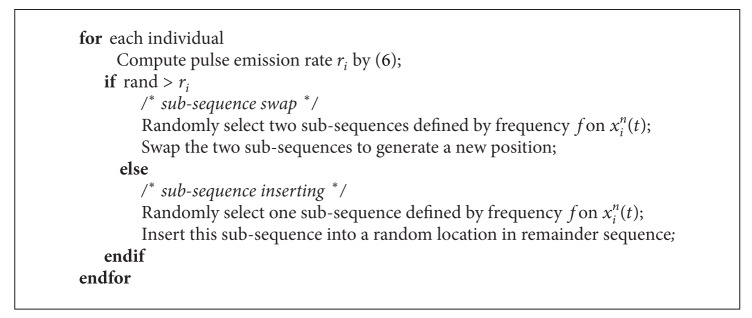
The pseudocode of pulse emission rate local operation.

**Algorithm 4:**
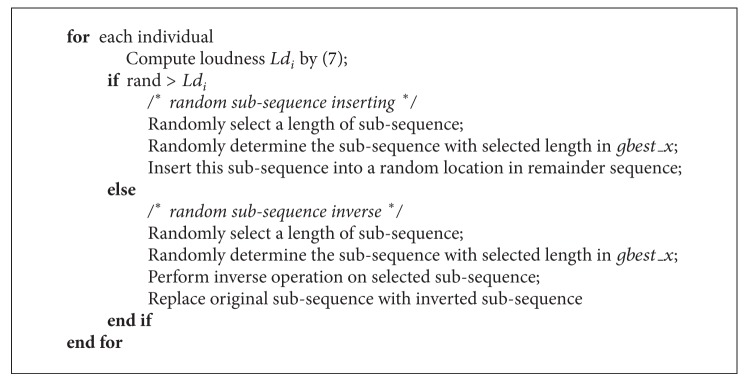
The pseudocode of loudness local operation.

**Algorithm 5:**
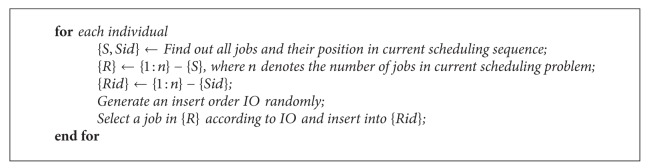
The pseudocode of adjustment.

**Algorithm 6:**
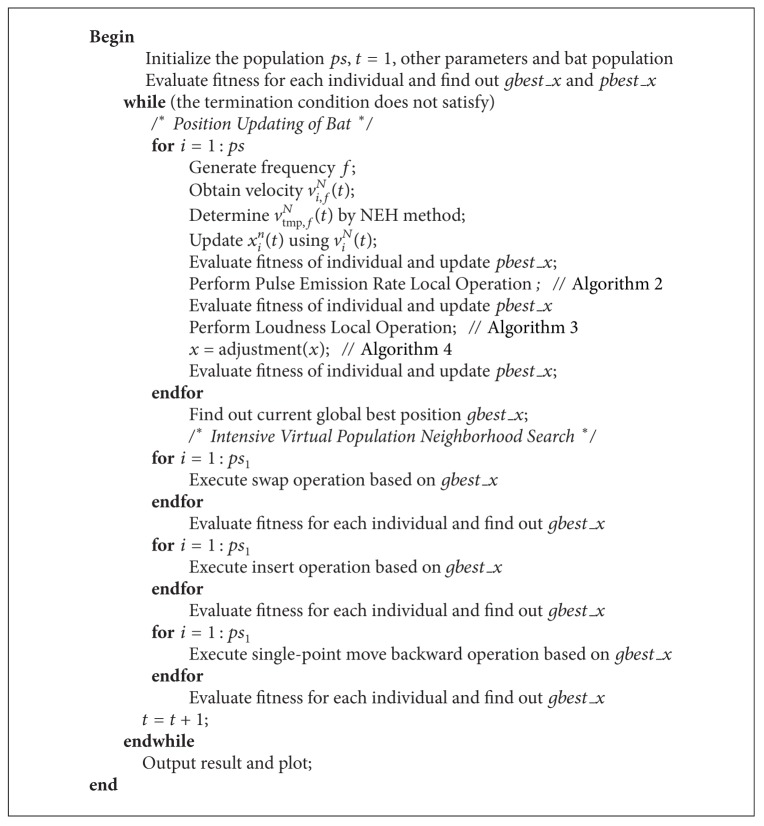
The DBA for PFSP.

**Table 1:** Statistical performances of DBA, DBA_NEH1, and DBA-IVPNS.

Problem	*n*∣*m*	C*	DBA				DBA_NEH1				DBA-IVPNS			
			BRE	ARE	WRE	Std	BRE	ARE	WRE	Std	BRE	ARE	WRE	Std
Car1	11∣5	7038	**0**	**0**	**0**	**0**	**0**	**0**	**0**	**0**	**0**	**0**	**0**	**0**
Car2	13∣4	7166	**0**	0.195	2.931	54.22	**0**	0.391	2.931	73.89	**0**	**0**	**0**	**0**
Car3	12∣5	7312	**0**	0.476	**1.190 **	44.12	**0**	0.635	**1.190 **	44.93	**0**	**0.397 **	**1.190 **	42.45
Car4	14∣4	8003	**0**	**0**	**0**	**0**	**0**	**0**	**0**	**0**	**0**	**0**	**0**	**0**
Car5	10∣6	7720	**0**	**0.246 **	**1.308 **	**35.70 **	**0**	0.664	1.360	45.95	**0**	0.352	**1.308 **	40.88
Car6	8∣9	8505	**0**	**0**	**0**	**0**	**0**	**0**	**0**	**0**	**0**	**0**	**0**	**0**
Car7	7∣7	6590	**0**	**0**	**0**	**0**	**0**	**0**	**0**	**0**	**0**	**0**	**0**	**0**
Car8	8∣8	8366	**0**	**0**	**0**	**0**	**0**	**0**	**0**	**0**	**0**	**0**	**0**	**0**
Rec1	20∣5	1247	**0.160 **	**0.209 **	**0.722 **	**1.84 **	**0.160 **	0.241	1.043	2.85	**0.160 **	0.545	1.925	7.92
Rec3	20∣5	1109	0.090	0.481	2.164	7.09	**0**	0.499	1.803	6.09	0.180	**0.385 **	1.713	4.51
Rec5	20∣5	1242	**0.242 **	**0.623 **	**2.174 **	9.49	**0.242 **	0.768	2.496	10.72	**0.242 **	1.100	2.496	10.69
Rec7	20∣10	1566	**1.149 **	1.443	**3.831 **	**11.41 **	**1.149 **	2.048	**3.831 **	18.33	**1.149 **	**1.537 **	**3.831 **	13.54
Rec9	20∣20	1537	**0**	2.420	3.709	13.44	**0**	**2.065 **	**3.318 **	15.98	1.041	2.728	4.815	13.13
Rec11	20∣10	1431	0.559	1.975	**3.564 **	**13.56 **	**0**	2.241	7.617	29.69	**0**	**1.859 **	4.403	16.64
Rec13	20∣15	1930	**0.415 **	2.394	3.938	19.84	0.933	2.525	4.819	19.30	1.762	2.694	4.352	16.29
Rec15	20∣15	1950	**0.154 **	**2.178 **	4.615	23.43	0.821	2.410	4.615	24.23	1.231	2.903	**4.256 **	**19.21 **
Rec17	20∣15	1902	0.946	**2.685 **	**4.206 **	**19.27 **	**0.894 **	3.582	5.941	25.20	1.577	5.065	6.730	25.65
Rec19	30∣10	2093	**0.573 **	2.621	4.252	21.38	1.386	**2.599 **	4.730	19.58	2.484	3.883	5.542	20.28
Rec21	30∣10	2017	**1.438 **	**2.310 **	**4.412 **	19.89	1.636	2.568	5.702	24.05	1.785	3.543	5.255	19.50
Rec23	30∣10	2011	**0.945 **	3.216	5.868	23.88	1.591	**3.090 **	4.923	19.22	3.282	4.422	6.266	18.48
Rec25	30∣15	2513	2.348	3.520	5.213	20.71	**1.870 **	**3.489 **	**5.133 **	23.77	3.780	5.428	6.805	**19.57 **
Rec27	30∣15	2373	2.402	3.638	**5.057 **	**19.03 **	**1.728 **	**3.217 **	5.900	23.66	2.023	4.374	5.942	23.93
Rec29	30∣15	2287	**1.530 **	4.323	7.084	33.64	2.186	**3.615 **	**5.597 **	24.60	4.766	6.046	7.521	**19.20 **
Rec31	50∣10	3045	3.284	**4.917 **	**6.502 **	30.44	**3.153 **	4.926	6.765	38.21	5.353	6.192	7.783	**21.95 **
Rec33	50∣10	3114	**0.835 **	**1.916 **	4.143	29.20	1.317	2.338	4.528	26.65	1.927	2.899	4.689	**25.99 **
Rec35	50∣10	3277	0.092	0.484	2.014	18.73	0.092	1.082	3.021	36.89	0.244	1.107	2.563	20.99
Rec37	75∣20	4951	5.615	**7.172 **	**8.140 **	39.66	5.918	7.387	8.826	37.75	8.503	9.156	10.261	**21.68 **
Rec39	75∣20	5087	3.696	**5.578 **	**6.408 **	41.52	4.914	6.083	7.529	35.22	6.979	7.629	8.374	**23.50 **
Rec41	75∣20	4960	**6.129 **	**7.435 **	**8.952 **	33.55	6.573	7.589	**8.952 **	**29.22 **	8.105	9.319	10.726	37.80

Average			**1.124 **	**2.154 **	**3.531 **	20.17	1.261	2.278	3.882	22.62	1.951	2.881	4.095	**16.68 **

**Table 2:** Statistical performances of DBA, PSOMA, PSOVNS, and OSA.

Problem	DBA					PSOVNS			PSOMA			OSA		
	*C*_*max*_	BRE	ARE	WRE	Std	BRE	ARE	WRE	BRE	ARE	WRE	BRE	ARE	Std
Car1	7038	0	0	0	0	0	0	0	0	0	0	0	0	0
Car2	7166	0	0	0	0	0	0	0	0	0	0	0	0	0
Car3	7312	0	0.397	1.190	42.45	0	0.420	1.189	0	0	0	0	0.625	47.19
Car4	8003	0	0	0	0	0	0	0	0	0	0	0	0	0
Car5	7720	0	0	0	0	0	0.039	0.389	0	0.018	0.375	0	0.801	50.73
Car6	8505	0	0	0	0	0	0.076	0.764	0	0.114	0.764	0	2.093	274.71
Car7	6590	0	0	0	0	0	0	0	0	0	0	0	1.483	114.21
Car8	8366	0	0	0	0	0	0	0	0	0	0	0	2.297	254.63
Rec1	1247	0	0.080	0.160	0.85	0.160	0.168	0.321	0	0.144	0.160	0.160	0.160	0
Rec3	1109	0	0.081	0.180	0.88	0	0.158	0.180	0	0.189	0.721	0	0.189	1.85
Rec5	1245	0.242	0.242	0.242	0	0.242	0.249	0.420	0.242	0.249	0.402	0.242	0.588	4.62
Rec7	1566	0	0.575	1.149	9.40	0.702	1.095	1.405	0	0.986	1.149	0	0.434	11.59
Rec9	1537	0	0.638	2.407	15.00	0	0.651	1.366	0	0.621	1.691	0	0.690	12.39
Rec11	1431	0	1.167	2.655	11.17	0.071	1.153	2.656	0	0.129	0.978	0	2.215	37.60
Rec13	1938	0.415	1.461	3.782	19.01	1.036	1.790	2.643	0.259	0.893	1.502	0.311	1.793	14.69
Rec15	1953	0.154	1.226	2.103	7.97	0.769	1.487	2.256	0.051	0.628	1.076	0.718	1.569	16.07
Rec17	1909	0.368	1.277	2.154	41.65	0.999	2.453	3.365	0	1.330	2.155	1.840	3.796	36.72
Rec19	2105	0.573	0.929	2.023	33.06	1.529	2.099	2.532	0.430	1.313	2.102	0.287	0.803	9.48
Rec21	2046	1.438	1.671	2.231	4.04	1.487	1.671	2.033	1.437	1.596	1.636	1.438	1.477	1.69
Rec23	2027	0.796	1.173	2.381	39.27	1.343	2.106	2.884	0.596	1.310	2.038	0.497	0.854	10.82
Rec25	2554	1.632	2.921	3.940	18.96	2.388	3.166	3.780	0.835	2.085	3.233	1.194	1.938	15.06
Rec27	2397	1.011	1.419	2.298	21.35	1.728	2.463	3.203	1.348	1.605	2.402	0.843	1.845	21.06
Rec29	2311	1.049	2.580	3.935	22.84	1.968	3.109	4.067	1.442	1.888	2.492	0.612	2.882	38.83
Rec31	3115	2.299	3.392	4.532	23.66	2.594	3.232	4.237	1.510	2.254	2.692	0.296	1.333	30.39
Rec33	3133	0.610	0.728	1.734	39.40	0.835	1.007	1.477	0	0.645	0.834	0.128	0.732	7.32
Rec35	3277	0	0.037	0.092	1.52	0	0.038	0.092	0	0	0	0	0	0
Rec37	5118	3.373	4.872	5.979	40.31	4.383	4.949	5.736	2.101	3.537	4.039	2.000	2.751	25.43
Rec39	5203	2.280	3.851	5.347	45.97	2.850	3.371	5.585	1.553	2.426	2.830	0.767	1.240	12.31
Rec41	5149	3.810	5.095	6.532	42.89	4.173	4.867	5.585	2.641	3.684	4.052	1.734	2.726	39.38

**Table 3:** Optimal job permutations of DBA.

Problem	*n*∣*m*	C*	*π*_∗_
Car1	11∣5	7038	8, 1, 3, 11, 5, 9, 4, 10, 7, 2, 6
Car2	13∣4	7166	7, 3, 4, 11, 9, 1, 8, 12, 5, 2, 13, 10, 6
Car3	12∣5	7312	11, 6, 5, 10, 12, 9, 3, 2, 4, 7, 8, 1
Car4	14∣4	8003	4, 12, 13, 14, 5, 7, 6, 1, 9, 10, 11, 8, 2, 3
Car5	10∣6	7720	5, 4, 2, 1, 3, 8, 6, 10, 9, 7
Car6	8∣9	8505	7, 1, 5, 6, 8, 3, 4, 2
Car7	7∣7	6590	5, 4, 2, 6, 7, 3, 1
Car8	8∣8	8366	7, 3, 8, 5, 2, 1, 6, 4
Rec1	20∣5	1247	6, 9, 2, 20, 12, 14, 17, 15, 13, 7, 1, 18, 3, 4, 11, 5, 8, 10, 19, 16
Rec3	20∣5	1109	6, 14, 7, 1, 2, 3, 11, 8, 9, 17, 15, 5, 19, 4, 16, 10, 12, 13, 18, 20
Rec7	20∣10	1566	17, 13, 18, 12, 9, 1, 6, 3, 8, 4, 5, 2, 7, 15, 10, 19, 11, 16, 14, 20
Rec9	20∣20	1537	4, 19, 17, 12, 18, 14, 7, 16, 5, 13, 2, 10, 9, 11, 8, 20, 1, 15, 3, 6
Rec11	20∣10	1431	16, 2, 14, 9, 12, 4, 20, 13, 10, 19, 8, 11, 3, 5, 15, 17, 1, 18, 7, 6
Rec35	50∣10	3277	13, 14, 40, 39, 50, 36, 46, 35, 37, 26, 2, 18, 19, 8, 41, 10, 25, 20, 38, 29, 33, 15, 27, 9, 21, 17, 42, 22, 32, 3, 1, 23, 4, 12, 5, 49, 11, 45, 43, 16, 34, 6, 44, 30, 7, 48, 47, 28, 24, 31

**Table 4:** The statistical results of score.

Benchmark	DBA				PSOVNS			PSOMA			SGA + NEH		OSA		
	BRE	ARE	WRE	Std	BRE	ARE	WRE	BRE	ARE	WRE	BRE	ARE	BRE	ARE	Std
Car1–Car8	32	31	30	32	32	27	28	32	29	30	30	16	32	19	27
Rec1–Rec41	60	58	62	70	40	37	56	73	66	78	20	4	73	57	77
Car1–Rec29	78	78	78	83	63	54	67	85	75	84	47	19	82	54	81
Car1–Rec41	92	89	92	102	72	64	84	105	95	108	50	20	105	76	104
